# Supplementary material for: Complexome profiling of the Chlamydomonas psb28 mutant reveals TEF5 as an early PSII assembly factor
Source: Plant Cell. 2025 Mar 17;37(6):koaf055. doi: 10.1093/plcell/koaf055 (PMC12164590; doi:10.1093/plcell/koaf055)
Supplement: koaf055_Supplementary_Data [file koaf055_supplementary_data.zip › psb28_interactive.zip!/plots/intensity/qualityPlots/peptides/_Cre07.g325736.t1.1.html]

### Cre07.g325736.t1.1

Trivial name:   
SeqCoverage [median]: 25.30  
Mass: 56.21 kDa  
  
 

| Sequence | MissedCleavage | Mass [Da] | wtIntensity | muIntensity | Score |
| --- | --- | --- | --- | --- | --- |
| AAIAAAAPAVAR | 0 | 1051.61 | 2176.51 | 2716.69 | 98.30 |
| AISQQLDAAAGAK | 0 | 1242.66 | 6738.53 | 3227.95 | 121.83 |
| AKVDEAEADIK | 1 | 1187.60 | 1010.00 | 359.07 | 42.09 |
| ATAVGGPLR | 0 | 840.48 | 1552.27 | 759.82 | 77.06 |
| KLESAATVAER | 1 | 1173.64 | 1716.46 | 1028.88 | 80.17 |
| SAGSGLKEVTAAR | 1 | 1245.67 | 765.63 | 0.00 | 60.63 |
| VAKDAVVAAEK | 1 | 1099.62 | 0.00 | 430.36 | 36.42 |
| VAVAPAAPAR | 0 | 921.54 | 4748.59 | 2756.63 | 79.12 |
| VAVAPPSRPGSGK | 1 | 1221.68 | 6474.88 | 4470.50 | 89.40 |
| VAVAPSR | 0 | 698.41 | 728.69 | 0.00 | 79.45 |
|  |  |  | 25911.6 | 15749.9 |  |
